# Cancer-Related Somatic Mutations in Transmembrane Helices Alter Adenosine A1 Receptor Pharmacology

**DOI:** 10.3390/molecules27123742

**Published:** 2022-06-10

**Authors:** Xuesong Wang, Willem Jespers, Kim A. N. Wolff, Jill Buytelaar, Adriaan P. IJzerman, Gerard J. P. van Westen, Laura H. Heitman

**Affiliations:** 1 Drug Discovery and Safety, Leiden Academic Centre for Drug Research, Einsteinweg 55, 2333 CC Leiden, The Netherlands; x.wang@lacdr.leidenuniv.nl (X.W.); w.jespers@lacdr.leidenuniv.nl (W.J.); kimwolff96@hotmail.com (K.A.N.W.); jill.buytelaar@gmail.com (J.B.); ijzerman@lacdr.leidenuniv.nl (A.P.I.); 2 Oncode Institute, 2333 CC Leiden, The Netherlands

**Keywords:** G protein-coupled receptors, adenosine A_1_ receptor, cancer, mutation, yeast system

## Abstract

Overexpression of the adenosine A_1_ receptor (A_1_AR) has been detected in various cancer cell lines. However, the role of A_1_AR in tumor development is still unclear. Thirteen A_1_AR mutations were identified in the Cancer Genome Atlas from cancer patient samples. We have investigated the pharmacology of the mutations located at the 7-transmembrane domain using a yeast system. Concentration–growth curves were obtained with the full agonist CPA and compared to the wild type hA_1_AR. H78L^3.23^ and S246T^6.47^ showed increased constitutive activity, while only the constitutive activity of S246T^6.47^ could be reduced to wild type levels by the inverse agonist DPCPX. Decreased constitutive activity was observed on five mutant receptors, among which A52V^2.47^ and W188C^5.46^ showed a diminished potency for CPA. Lastly, a complete loss of activation was observed in five mutant receptors. A selection of mutations was also investigated in a mammalian system, showing comparable effects on receptor activation as in the yeast system, except for residues pointing toward the membrane. Taken together, this study will enrich the view of the receptor structure and function of A_1_AR, enlightening the consequences of these mutations in cancer. Ultimately, this may provide an opportunity for precision medicine for cancer patients with pathological phenotypes involving these mutations.

## 1. Introduction

G protein-coupled receptors (GPCRs) are the largest protein superfamily in the human genome with approximately 800 subtypes [1]. They share structural characteristics of seven-transmembrane helices (TMs) connected by an extracellular N-terminus, three extracellular loops (ELs), three intracellular loops (ILs), and an intracellular C-terminus [2]. GPCRs are widely distributed throughout the human body and regulate various crucial cellular and physiological functions by responding to a diverse set of endogenous ligands [3]. However, their aberrant activity and expression also substantially contribute to human pathophysiology [4].

Kinases, due to their central roles in the cell cycle, have been studied as a primary focus in preclinical oncology over the last two decades [5]. GPCRs, however, have been relatively under-investigated in this context, while an increasing amount of evidence shows that GPCRs act as regulators of tumor initiation and progression [6]. Malignant cells often hijack the normal physiological functions of GPCRs to survive, invade surrounding tissue, and evade the immune system [7]. Moreover, somatic mutations of GPCRs have been identified in approximately 20% of all cancers by a systematic analysis of cancer genomes [5]. 

The immune system plays a fundamental and essential role in the defense against cancer [8]. Adenosine, a nucleoside, and derivative of ATP, has emerged as a major immune–metabolomic checkpoint in tumors [9]. Compared to healthy tissue, adenosine is accumulated over 50-fold in the hypoxic tumor environment, leading to a reduced anti-tumoral immune response [10]. Adenosine regulates various physiological effects and immune responses in cancer via adenosine receptors (ARs): the A_1_, the A_2A_, the A_2B_, and the A_3_ receptor [11]. Additionally, all ARs have been detected in different human tumor tissues [12]. Therefore, all four subtypes of ARs may regulate cancer progression in one way or another. 

Growing evidence addresses the involvement of A_1_AR in cancer progression, although its precise role is not well understood [13,14]. An increased expression level of the A_1_AR has been detected in diverse cancer cells [15,16], where it appears to behave as both an anti- and pro-tumoral regulator in the development of different cancer types [10]. Interestingly, various single-site point mutations on A_1_AR have been isolated from patients with different cancer types and collected by the TCGA Research Network (https://www.cancer.gov/tcga, 24 February 2022). Previous site-directed mutagenesis and docking studies on A_1_AR have identified residues all over the protein involved in ligand recognition and/or functional activity [17,18]. Furthermore, several GPCR-conserved residues and motifs, for instance, the D2.50 residue, the ionic lock, the NPxxY motif, and the DRY motif, are located at 7-TM domains mediating ligand binding and signaling [19]. 

In this study, 13 mutations located at the 7-TM domains of the A_1_AR have been selected from cancer patients using a bioinformatics approach. The effects of these mutant receptors on constitutive receptor activity and agonist-induced activation were tested in a ‘single-GPCR-one-G protein’ *S. cerevisiae* strain, which has been reported to be predictive of the mammalian situation [20,21]. Selected mutant receptors were further investigated for their effects on ligand binding and receptor activation in a mammalian system. Subsequently, we identified two CAMs, five CIMs, and six loss-of-function mutants (LFMs) based on the pharmacological effects of these mutant receptors. Thus, cancer-related mutations within the 7-TM domain may alter the role of A_1_AR in cancer progression and the efficacy of drugs targeting A_1_AR as a cancer therapeutic approach.

## 2. Results

### 2.1. Data Mining

**Figure 1:**
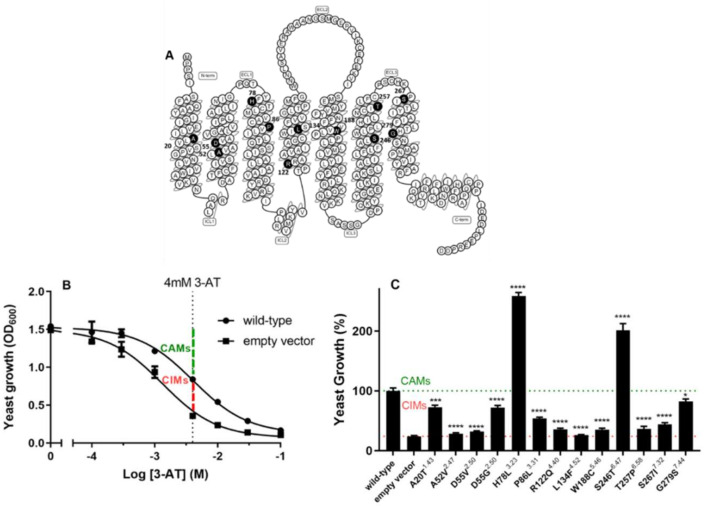
(**A**) Snake plot of wild type hA_1_AR. Mutated residues are marked in black. (**B**) Concentration–growth curves of the yeast strain in the presence or absence of wild type hA_1_AR. The combined graph is shown as mean ± SEM from three individual experiments performed in duplicate. (**C**) Constitutive activity of wild type and 13 mutant hA_1_ARs in the presence of 4 mM 3-AT. The yeast growth with wild type hA_1_AR was set to 100% and the background of the selection medium was set to 0%. The bar graph is the combined result of three independent experiments performed in quadruplicate. * *p* < 0.05; *** *p* < 0.001; **** *p* < 0.0001 compared to wild type hA_1_AR, determined by using one-way ANOVA with Dunnett’s post-test. CAM: constitutively active mutant, CIM: constitutively inactive mutant.

Mutation data from cancer patient isolates were obtained by data mining the TCGA database on August 8, 2015. A total of 27 point somatic mutations were selected from (in total) 48 cancer-related point mutations of hA_1_ARs based on selected cancer types, i.e., breast invasive carcinoma, colon adenocarcinoma, lung adenocarcinoma, lung squamous cell carcinoma, lymphoid neoplasm diffuse large B-cell lymphoma and rectum adenocarcinoma. After assigning Ballesteros Weinstein numbers to the positions by using the GPCRdb alignment tool, 13 mutations located at the 7-TM domains were selected for this study (Table 1). One mutation was located at the first, two at the second, two at the third, one at the fourth, one at the fifth, two at the sixth, and two at the seventh TM (Figure 1A).

### 2.2. Constitutive Activity of Mutant hA_1_ARs

To first characterize the effect of the cancer-related mutations on the constitutive activity of the receptor, i.e., activity independent from an agonist, yeast growth assays were performed in the absence of the agonist. First, the optimal concentration of the histidine biosynthesis inhibitor (3-amino-1,2,4-triazole, 3-AT) for constitutive activity screening was determined in response to increasing concentrations of 3-AT (Figure 1B). Upon increasing concentrations of 3-AT, cell growth of both yeast cells transformed with a plasmid, with or without wild type hA_1_AR, decreased (Figure 1B). At a concentration of 4 mM 3-AT, the two curves showed the largest differences in yeast growth; at this point, mutant receptors with increased constitutive activity (CAM) would show a higher growth level than wild type hA_1_AR, while mutant receptors with decreased constitutive activity (CIM) would show a growth level in between wild type hA_1_AR and empty vector. Thus, using this concentration of 3-AT provided the best window to screen for both CAMs and CIMs.

Cancer-related mutations showed various effects on the constitutive activity of the hA_1_AR (Figure 1C). Eleven out of the thirteen mutant receptors had a decreased constitutive activity compared to the wild type hA_1_AR. Among them, mutant receptors A52V^2.47^, D55V^2.50^, R122Q^4.40^, L134F^4.52^, W188C^5.46^, and T257P^6.58^ even showed similar activities as yeast cells transformed by the empty vector. In contrast, increased constitutive activity was observed on two mutant receptors, i.e., H78L^3.23^ and S246T^6.47^. 

### 2.3. Agonist-Induced Receptor Activation of Mutant hA_1_ARs

**Figure 2:**
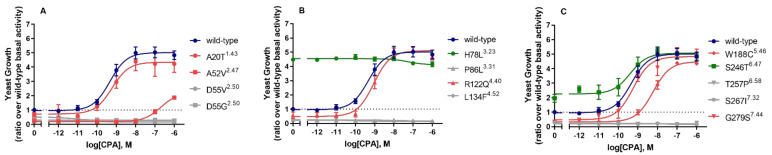
Concentration–response curves of wild type and mutated hA_1_ARs. Data are separated for mutations located on (**A**) the first and second transmembrane helix, (**B**) third and fourth transmembrane helix, and (**C**) fifth, sixth, and seventh transmembrane helix. Data were normalized as ratio over basal activity of wild type hA_1_AR (dotted line). Combined graphs are shown as mean ± SEM from at least three individual experiments performed in duplicate. Data for wild type are shown in dark blue, for CIMs in red, for CAMs in green, and for LFMs in grey.

To further characterize the activation profiles of these mutations, concentration–growth curves were determined in the presence of increasing concentrations of the selective hA_1_AR full agonist, CPA, and 7 mM 3-AT (Figure 2 and Table 2). Wild type hA_1_AR showed a potency/pEC_50_ value of 9.30 ± 0.08 and a maximum effect/E_max_ value (ratio over wild type basal activity) of 4.83 ± 0.30 in the yeast system (Table 2).

Almost half of the mutant receptors with decreased constitutive activity could not be activated by CPA anymore, namely D55V^2.50^, D55G^2.50^, P86L^3.31^, L134F^4.52^, T257P^6.58^, and S246I^7.32^, which resulted in typing them as loss of function mutants (Figure 2 and Table 2). Other mutant receptors with decreased constitutive activity could still be activated by CPA with equal or lower potency and efficacy values. Specifically, in response to CPA, mutant receptors A20T^1.43^, R122Q^4.40^, and G279S^7.44^ were activated to a similar activation level as wild type hA_1_AR with pEC_50_ values of 9.24 ± 0.08, 9.04 ± 0.14, and 9.27 ± 0.09, which were also not significantly different from the pEC_50_ value of the wild type receptor. Mutant receptor A52V^2.47^ had a much lower efficacy (1.86 ± 0.14) in the presence of 1 µM of CPA than wild type hA_1_AR, and also showed a more than 400-fold decreased potency. The activation level of mutant receptor W188C^5.46^ was similar to wild type hA_1_AR (4.35 ± 0.10), while the potency of CPA was decreased by 10-fold.

The two mutant receptors with increased constitutive activity, namely H78L^3.23^ and S246T^6.47^, also showed increased constitutive activity in concentration–growth curves. In response to CPA, mutant receptor S246T^6.47^ was activated to a similar E_max_ level (4.81 ± 0.26) with a similar pEC_50_ value for CPA (9.42 ± 0.33) as at the wild type hA_1_AR (Figure 2C and Table 2). Interestingly, mutant receptor H78L^3.23^ showed a 4.5-fold increase in constitutive activity compared to wild type, where further activation could not be obtained anymore by the addition of CPA (Figure 2B and Table 2).

**Figure 3:**
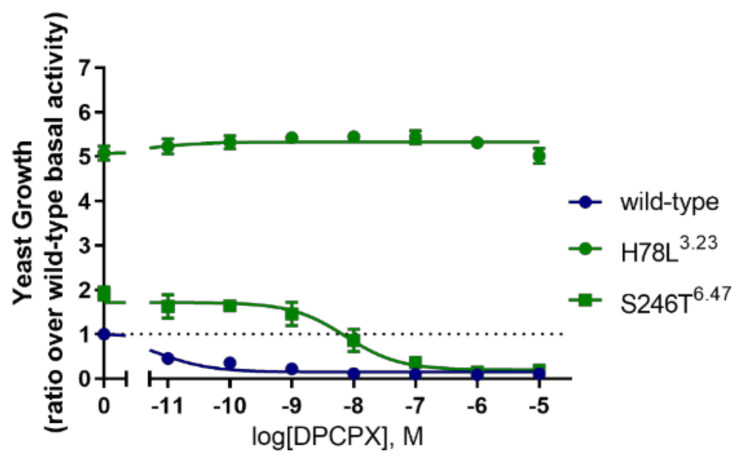
Concentration–inhibition curves of the hA_1_AR inverse agonist DPCPX at the wild type A_1_AR and the CAMs, H78L^3.23^, and S246T^6.47^. Data were normalized as ratio over basal activity of wild type hA_1_AR (dotted line). Combined graphs are shown as mean ± SEM from at least three individual experiments performed in duplicate. Data for wild type are shown in dark blue and for CAMs in green.

Next, we investigated whether the increased constitutive activity of these two mutants could be decreased using an inverse agonist, DPCPX (Figure 3). For mutant receptor S246T^6.47^, DPCPX reduced the constitutive activity to wild type hA_1_AR levels with a pIC_50_ value of 8.55 ± 0.25. However, the high constitutive activity of mutant receptor H78L^3.23^ was not reduced by DPCPX. 

Evaluating the diverse pharmacological effects of these mutant receptors, we characterized mutant receptors H78L^3.23^ and S246T^6.47^ as CAMs, mutant receptors A20T^1^.^43^, A52V^2.47^, R122Q^4.40^, W188C^5.46^, and G279S^7.44^ as CIMs, and mutant receptors D55V^2.50^, D55G^2.50^, P86L^3.31^, L134F^4.52^, T257P^6.58^, and S267I^7.32^ as loss of function mutants (LFMs) (Table 2).

### 2.4. Ligand Binding on Wild Type and Mutant hA_1_ARs

**Figure 4:**
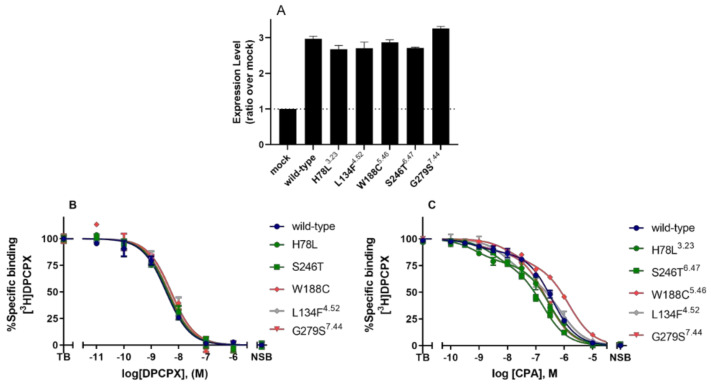
(**A**) Cell surface expression levels of wild type and mutant hA_1_AR transiently transfected on CHO cell membranes, as determined by ELISA. Data were normalized as ratio over mock-transfected CHO cells (mock, dotted line) and shown as mean ± SEM obtained from three individual experiments performed in pentaplicate. (**B**,**C**) Displacement of specific [^3^H]DPCPX binding to the transiently transfected wild type hA_1_AR, LFM L134F^4^^.52^, CIMs W188C^5^^.46^ and G279S^7^^.44^, and CAMs H78L^3.23^ and S246T^6.47^ on CHO cell membranes by DPCPX and CPA, respectively. Combined graphs are shown as mean ± SEM from three individual experiments, each performed in duplicate. Data for wild type are shown in dark blue, for CIMs shown in red, for CAMs in green, and for LFMs in grey.

Selected mutants with diverse effects on receptor activation, i.e., H78L^3^^.23^, L134F^4^^.52^, W188C^5^^.46^, S246T^6^^.47^, and G279S^7^^.44^, were further investigated on ligand binding in a mammalian expression system. Wild type and mutant receptors were transiently transfected into Chinese Hamster Ovary (CHO) cells, and receptor expression levels were measured by ELISA. All mutant receptors were expressed on the cell surface with similar levels to the wild type hA_1_AR (Figure 4A). 

Affinity values of the radioligand [^3^H]DPCPX and B_max_ values of wild type and mutant hA_1_ARs were determined by homologous competition displacement assays on transiently transfected membranes (Figure 4 and Table 3). [^3^H]DPCPX had a pK_D_ value of 8.36 ± 0.03 at the wild type hA_1_AR, which was significantly higher than the value on LFM L134F^4^^.52^ (8.06 ± 0.08), but lower than the value on CIM G279S^7^^.44^ (8.62 ± 0.06, Table 3). Mutant receptors H78L^3^^.23^, W188C^5^^.46^, and S246T^6^^.47^ showed similar pK_D_ values of [^3^H]DPCPX compared to the wild type hA_1_AR. Diverse B_max_ values were obtained on mutant receptors in comparison to wild type hA_1_AR (1.18 ± 0.14 pmol/mg). A significantly increased expression level of 3.74 ± 0.65 pmol/mg was observed on LFM L134F^4.52^, while expression levels of CAMs H78L^3.23^ and S246T^6.47^ were decreased (0.17 ± 0.01 pmol/mg and 0.11 ± 0.01 pmol/mg). Note that these values did not correlate with the cell surface expression data obtained from ELISA.

Heterologous displacement by CPA of [^3^H]DPCPX radioligand binding on all mutant receptors as well as wild type hA_1_AR was best fitted to a two-site model (Figure 4C and Table 3). Wild type hA_1_AR had a pK_i_ value of 9.24 ± 0.26 for the high-affinity state, 6.76 ± 0.05 for the low-affinity state with a fraction value of 0.15 ± 0.03 for the high-affinity state. Decreased pK_i_ values were observed on CIM W188C^5.46^ for both high and low-affinity states (8.02 ± 0.16 at high affinity state and 6.15 ± 0.01 at low-affinity state). LFM L134F^4.52^ also showed a decreased affinity value of 6.26 ± 0.11 at the low-affinity state compared to the wild type receptor, while the high-affinity state was unchanged. Lastly, CAM S246T^6.47^ had an increased affinity value of 7.19 ± 0.08 at the low-affinity state with an unaffected affinity in the high-affinity state.

### 2.5. [^35^S]GTPγS Functional Assay on Wild Type and Mutant hA_1_ARs

**Figure 5:**
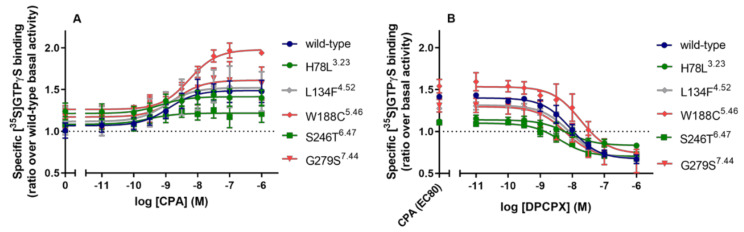
[^35^S]GTPγS binding to the transiently transfected wild type hA_1_AR, LFM L134F^4.52^, CIMs W188C^5.46^ and G279S^7.44^, and CAMs H78L^3.23^ and S246T^6.47^ on CHO cell membranes. (**A**) Receptor activation of wild type and mutant hA_1_ARs stimulated by CPA. Data were normalized as ratio over basal activity of wild type hA_1_AR. (**B**) Concentration–inhibition curves of DPCPX with the presence of CPA at the concentration of EC_80_ for wild type and mutant hA_1_AR. Data were normalized as ratio over basal activity of wild type or mutant hA_1_AR. Data were obtained from three different experiments, each performed in duplicate. Data for CIMs are shown in red, for CAMs in green, and for LFMs in grey.

CHO cell membranes transiently transfected with wild type and mutant hA_1_AR were further tested in a functional assay, i.e., GTPγS binding (Figure 5 and Table 4). All selected mutant receptors showed similar basal activity to wild type hA_1_AR. In response to CPA, wild type hA_1_AR showed a potency/pEC_50_ value of 8.98 ± 0.08 and an E_max_ value (ratio over wild type basal activity) of 1.48 ± 0.13. Only CIM W188C^5.46^ showed altered receptor pharmacology upon activation by CPA with a decreased potency value of 8.28 ± 0.10, while the efficacy was not significantly affected. While LFM L134F^4.52^ did not show any activation in the yeast system, it could be activated in the mammalian system with similar potency and efficacy values for CPA compared to wild type. CAM S246T^6.47^ showed altered receptor pharmacology upon CPA-mediated activation with a higher pEC_50_ value of 9.44 ± 0.22 and a slightly lower efficacy value of 1.21 ± 0.10 than wild type hA_1_AR, albeit not significantly different. CIM G279S^7.44^ did not show significantly different receptor pharmacology to wild type hA_1_AR in the mammalian system.

Next, we investigated whether the agonist-mediated activation could be inhibited by the antagonist, DPCPX, on wild type and mutant receptors (Figure 5B). For the wild type receptor, the activation level was reduced to 0.67 ± 0.05 with a pIC_50_ value of 8.09 ± 0.16 for DPCPX. In the mammalian system, the CPA-mediated activation for all mutant receptors was reduced to wild type levels with similar pIC_50_ values (Table 4). 

### 2.6. Structural Mapping and Bioinformatics Analysis of Mutations

**Figure 6:**
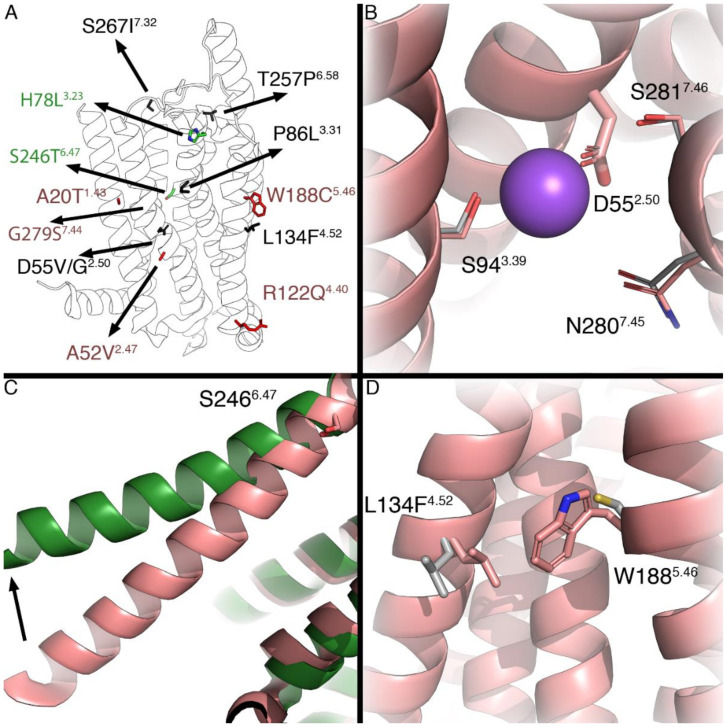
(**A**) Overview of all mutations mapped on the X-ray structure of the hA_1_AR, inactive (5UEN) in red and active (6D9H) in green. Residues are colored by their observed effect, CAMs in green, CIMs in red, and LFMs in black. (**B**) Close up of residue D55^2.50^. In grey, residues are found in the A_2A_AR binding site, with the sodium ion from that structure (PDB: 4EIY) in purple. (**C**) Residue S246^6.47^ is found near the hinging region of TM6, the outward motion of which is associated with receptor activation (shown with arrow). (**D**) Residues L134^4.52^ and W188^5.46^ form a cluster and are pointing toward the membrane.

The mutations investigated in this study were mapped on the inactive (5UEN) and active (6D9H) hA_1_AR structure to provide structural hypotheses for the observed pharmacological effects (i.e., CIM, CAM, and LFM) of the different mutations, and explain differences between yeast and mammalian data. Mutations were found scattered over the receptor structure, with LFMs indicated in black, CIMs in red, and CAMs in green (Figure 6A). Whilst some LFMs can be considered drastic changes (for instance, T257P^6.58^ and P86L^3.31^), others are relatively mild from a structural perspective (e.g., S267I^7.32^). LFMs D55V/G^2.50^ sit in the sodium ion binding pocket in direct contact with the sodium ion (Figure 6B). The CAM S246T^6.47^ is found near the middle of helix 6, which undergoes a large conformational change upon the receptor activation (Figure 6C). Finally, W188C^5.46^ and L134F^4.52^ are positioned close to one another and point toward the membrane. 

## 3. Discussion

Although the role of hA_1_AR in cancer progression remains unclear, a growing amount of studies suggest that hA_1_AR is involved in cancer development [13,14]. Previous structural studies and crystal structures of hA_1_AR provided us with information on crucial residues for ligand binding and receptor activation, as well as essential interactions in the inactive receptor state and G protein coupling [17,23,24,25]. Moreover, compared to other residues, accumulation of cancer-related mutations has been observed in highly conserved residues of the TM domains [26]. Therefore, in this study, we studied 13 single-site point mutations located at the 7-TM domains of A_1_AR obtained from The Cancer Genome Atlas (TCGA). All mutations were examined in the *S. cerevisiae* system and selected mutations were further investigated in the mammalian system to improve our understanding of the mechanism of receptor activation with respect to cancer development and progression.

### 3.1. Mutations Located at the Top Part of the Receptor

Mutant receptors H78L^3.23^, P86L^3.31^, T257P^6.58^, and S267I^7.32^, located at the top, extracellular part of the receptor, all showed dramatic changes upon receptor activation in the yeast system. Mutant receptor H78L^3.23^ showed an extremely high constitutive activity, which could not be further induced by CPA or reduced by DPCPX (Figure 2C and Figure 3 and Table 2). Although this could not be confirmed in the mammalian system (probably due to its low expression level), it indicates that H78L^3.23^-hA_1_AR is locked in an active conformation, which has been described previously on mutant receptor G14T^1.37^ in hA_1_AR [27]. Similar expression levels were not observed between ELISA and homologous competition assays (Figure 4A and Table 3) due to different experimental setups that whole-cell expressions of functioning receptors were determined in homologous competition assays [28]. Crystallographic structural evidence of the inactive state A_1_AR reveals that H78^3.23^ forms a salt bridge with E164, which is important for the stabilization of a β-sheet between EL1 and EL2 [24]. It is known that ELs are essential in ligand binding and the receptor activation mechanism in class A GPCR [18]. Therefore, we hypothesize that the loss of the anionic charge hinders the salt bridge formation and stabilizes the receptor conformation in its active state.

Mutant receptors P86L^3.31^, T257P^6.58^, and S267I^7.32^ were characterized as LFMs with complete loss of activation. This could be caused by loss of expression, while expression levels could not be determined in the yeast by a Western blot analysis due to the low selectivity of the primary antibody against hA_1_AR (data not shown). However, all mutant receptors of A_1_AR and A_2B_AR have previously been successfully expressed in the same yeast system, where differences in expression did not influence receptor activation [17,29]. It had been shown in a previous study on A_1_AR that mutant receptor P86F^3.31^ resulted in abolished CPA binding. This indicates that the proline at residue 86 indirectly affects ligand binding by reorienting the TM1 conformation to favor N^6^ substituents [30]. Both P86L^3.31^ and P86F^3.31^ are mutations in which the small size and rigid residue proline were exchanged by larger amino acids with hydrophobic side chains. The introduction of these larger side chains is potentially the causal factor for the loss of receptor activation. The residue T257^6.58^, located at the top part of the helix 6, forms a hydrophobic pocket along with M177^5.35^, L253^6.54^, and T270^7.35^_,_ which has been shown to accommodate the antagonist DU172 in the A_1_AR [24]. In A_2A_AR, an alanine mutation at residue T256^6.58^ has been shown to result in decreased affinity of the reference antagonist ZM241385 [31]. It is known that proline introduces kinks in α-helices due to the absence of an H-bond donor in addition to steric hindrance disrupting amide backbone hydrogen bond formation [32]. Therefore, in A_1_AR, the proline mutation at T257^6.58^ likely altered the receptor conformation and resulted in the loss of receptor activation. Mutant receptor S267I^7.32^, located at the top of helix 7 and end of ECL3, showed a complete loss of activation in response to CPA, indicating that residue S267 may indirectly affect ligand binding.

### 3.2. Mutations Located on Conserved Residues

Conserved residues and motifs of GPCRs are known to mediate ligand binding and receptor functionality [19]. Thus, mutations located at these residues may cause prominent alterations in receptor pharmacology. Alanine at residue 2.47 is highly conserved among class A GPCRs (72 %) [33]. Mutant receptor A52V^2.47^ showed a dramatic decrease in both potency and efficacy of CPA (Figure 2A and Table 2), which could not be confirmed in mammalian cells due to a lack of expression. Interestingly, this same mutation occurs in CCR5, where this seemingly small change in the side chain, has been reported to greatly affect the binding of CCL5 [34], indicating the essential role of residue A2.47 in receptor–ligand interaction. 

Two LFMs, D55G^2.50^ and D55V^2.50^, are found at residue D^2.50^, which is the most highly conserved residue among class A GPCRs (92%) [35]. D^2.50^ together with S^3.39^ regulates Na^+^-binding [36]. Mutations at residue D^2.50^ are known to alter ligand binding and/or G protein signaling [37,38]. Abolished G protein signaling has also been reported on mutant receptor D52N^2.50^ in A_2A_AR, in which it was shown that inter-helical packing was impacted by the change from aspartic acid to asparagine [37]. Therefore, our results implicate that the loss of the negatively-charged side chain in D^2.50^ impedes electrostatic interactions with Na^+^-ions and, thereby, leads to decreased receptor activation.

S246^6.47^ belongs to the conserved CWxP motif in helix 6, which is classified as the microswitch region and associated with receptor activation [39]. In the CWxP motif, cysteine at residue 6.47 is conserved by 71 % among class A GPCR and serine is 10% [35]. In both yeast and mammalian systems, mutant receptor S246T^6.47^ showed slightly increased potency values of CPA (Figure 2C, Figure 5A, Table 2 and Table 4). The increase in potency value could be caused by the increase in ligand binding of CPA (Figure 4C). Of note, the affinity of DPCPX was unchanged, implying that this antagonist has no preference for binding to the active or inactive state of the receptor [40]. Additionally, hA_1_AR was not locked in the active conformation by mutation S246T^6.47^, as DPCPX could still deactivate the receptor (Figure 3). Similarly, in the ß_2_-adrenergic receptor, the mutation C285T^6.47^ has been characterized as a CAM, while C285S^6.47^ had similar properties to the wild type receptor [39]. As it is known that residue 6.47 is crucial for the rotamer toggle switch [39], a threonine mutation on 6.47 may alter the side chain modulation of the rotamer toggle switch, further impacting the movement of TM6 during receptor activation. 

### 3.3. Mutations Located on Residues Pointing towards the Membrane

In mammalian cell membranes, cholesterol has been reported to have a modulatory role in the GPCR function via interaction with residues in the lipid–protein interface [41]. Moreover, compared to the membranes of mammalian cells, the yeast cell membrane contains less cholesterol and more ergosterol, which may result in a different receptor conformation and, thus, the functionality of human GPCRs between expression systems [41,42]. Moreover, the conflicting results obtained from different expression systems could be caused by differences in receptor expression levels. 

Mutant receptor G279S^7.44^ has been characterized as a CIM with retained potency and efficacy of CPA in the yeast system, while decreased constitutive activity could not be observed in the mammalian system, possibly due to the slightly higher expression level than wild type hA_1_AR. Interestingly, G279S^7.44^ has also been identified as a Parkinson’s disease-associated mutation, which did not alter receptor expression or ligand binding but influenced the heteromerization with the dopamine D_1_ receptor [43].

Mutant receptor W188C^5.46^ showed a 10-fold decrease in the potency value of CPA in both yeast and mammalian systems (Figure 2C, Figure 5A, Table 2 and Table 4). This decrease in potency was caused by the decrease in the affinity of CPA (Figure 4C and Table 3). Despite the maintenance of hydrophobicity of the side chain, the substitution of tryptophan for cysteine introduced a dramatic reduction in the side chain size. Reducing the amino acid side chain size at position W188^5.46^ may affect the receptor–ligand interaction of CPA on hA_1_AR. Moreover, it has been shown that W188^5^.^46^ together with residues V137^4.55^, F144^4.62^, W146, Y182^5.40^, F183^5.41^, and V187^5.45^ are part of a hydrophobic core, which, along with residues S150 and R154, forms contacts with the EL2 of two A_1_AR homodimers in mammalian cells [24]. It has been hypothesized that EL2 exerts a crucial role in the transition between G protein-coupled and -uncoupled states [44]. While it was previously suggested that A_1_AR homodimerizes, leading to cooperative orthosteric ligand binding in mammalian cells [45], the homodimerization of A_1_AR in yeast cells remains undetermined. 

Residue L134^4.52^ forms a cluster with W188^5.46^ pointing towards the membrane (Figure 6D). Mutant receptor L134F^4.52^ has been characterized as LFM in the yeast system. However, it behaved quite similar to wild type A_1_AR in the mammalian system (Figure 5 and Table 4). L134^4.52^ is conserved amongst all ARs and is located close to the highly conserved residue in TM4, W^4.50^. The latter is known to be involved in ligand binding and interaction with the cell membrane via cholesterol, where complete loss of ligand binding has been observed previously by mutating tryptophan to other amino acids [41,46,47]. Phenylalanine mutation at L134^4.52^ might thus indirectly change the interactions among residues W132^4.50^, L99^3.44^, A100^3.45^, L193^5.51^, and Y200^5.58^ [46], by the dramatic size change of the side chain, and this might be different when using a different cell membrane background.

### 3.4. Potential Role for hA_1_AR Mutations in Cancer

Activation of hA_1_AR has been identified with anti-proliferative effects in colon cancer, glioblastoma, and leukemia [10,48,49]. Mutations with inhibitory effects on receptor activation identified from colon cancer, such as the LFM D55G^2.50^ and CIM W188C^5.46^, might then behave as pro-proliferative regulators in cancer progression. In contrast, deletion or blockade of hA_1_AR resulted in inhibited cell proliferation but induced PD-L1 upregulation in melanoma cells, which led to compromised anti-tumor immunity [50]. Additionally, the hA_1_AR antagonist DPCPX shows inhibitory effects on tumor cell proliferation and migration while promoting apoptosis [12,15]. Mutant receptors with the altered binding affinity of DPCPX, namely L134F^4.52^ and W188C^5.46^ in this study, may thus impact the efficacy of DPCPX treatments. Of note, due to the low frequency in comparison to known driver mutations in cancer patients, these cancer-related mutations in hA_1_AR are unlikely to be cancer drivers [51]. However, passenger mutations should not be ruled out in consideration of cancer personalized therapy [52].

## 4. Materials and Methods

### 4.1. Data Mining

Mutation data were downloaded from The Cancer Genome Atlas (TCGA, version August 8, 2015; note, the TCGA data were frozen in early 2016) by using the Firehose tool [53]. MutSig 2.0 data were extracted when available, MutSig 2CV was used in cases where the former was not available (specifically for colon adenocarcinoma, acute myeloid leukemia, ovarian cerous cystadenocarcinoma, rectum adenocarcinoma). Natural variance data were downloaded from Uniprot (Index of Protein Altering Variants, version November 11 2015) [54]. Sequence data were filtered for missense somatic mutations and the A_1_AR (Uniprot identifier P30542). The GPCRdb alignment tool was used to assign Ballesteros Weinstein numbers [22,55] to the positions through which a selection could be made for transmembrane domain positions. 

### 4.2. Materials

The MMY24 strain and the *S. cerevisiae* expression vectors, the pDT-PGK plasmid and the pDT-PGK_hA_1_AR plasmid (i.e., expressing the wild type receptor) were kindly provided by Dr. Simon Dowell from GSK (Stevenage, UK). The pcDNA3.1(+) plasmid cloned with N-terminal 3xHA-tagged hA_1_AR was ordered from cDNA Resource Center (Bloomsburg, PA, USA). The QuikChange II^®^ Site-Directed Mutagenesis Kit containing XL10-Gold ultracompetent cells was purchased from Agilent Technologies (Amstelveen, The Netherlands). The QIAprep mini plasmid purification kit and QIAGEN^®^ plasmid midi kit were purchased from QIAGEN (Amsterdam, The Netherlands). Adenosine deaminase (ADA), 1,4-dithiothreitol (DTT), 8-cyclopentyl-1,3-dipropylxanthine (DPCPX), and 3-amino-[1,2,4]-triazole (3-AT) were purchased from Sigma-Aldrich (Zwijndrecht, The Netherlands). N^6^-cyclopentyladenosine (CPA) was purchased from Santa Cruz Biotechnology (Heidelberg, Germany). Bicinchoninic acid (BCA) and BCA protein assay reagent were obtained from Pierce Chemical Company (Rockford, IL, USA). Radioligands 1,3-[^3^H]-dipropyl-8-cyclopentylxanthine ([^3^H]DPCPX, specific activity of 137 Ci × mmol^−1^) and [^35^S]-Guanosine 5′-(γ-thio)triphosphate ([^35^S]GTPγS, a specific activity 1250 Ci × mmol^−1^) were purchased from PerkinElmer, Inc. (Waltham, MA, USA). Rabbit anti-HA antibody (71-5500) was purchased from Thermo Fisher Scientific (Waltham, MA, USA), while goat anti-rabbit IgG HRP was purchased from Jackson ImmunoResearch Laboratories (West Grove, PA, USA).

### 4.3. Generation of hA_1_AR Mutations

The plasmids carrying hA_1_AR mutations were constructed by polymerase chain reaction (PCR) mutagenesis as previously described, using pDT-PGK_hA_1_AR or pcDNA3.1_hA_1_AR with N-terminal 3xHA tag as the template [17]. The QuikChange Primer Design Program of Agilent Technologies (Santa Clara, CA, USA) was used to design primers for mutant receptors, and primers were purchased from Eurogentec (Maastricht, The Netherlands). All DNA sequences were verified by Sanger sequencing at LGTC (Leiden, The Netherlands).

### 4.4. Transformation in MMY24 S. Cerevisiae Strain

The plasmids, pDT-PGK_hA_1_AR, containing either wild type or mutated hA_1_AR were transformed into a MMY24 *S. cerevisiae* strain following the lithium acetate procedure [56].

### 4.5. Liquid Growth Assay

In order to characterize the mutant hA_1_ARs, liquid growth assays in 96-well plates were performed to obtain concentration–growth curves as previously described [17]. Briefly, yeast cells expressing wild type or mutant hA_1_AR were inoculated to 1 mL selective YNB medium lacking uracil and leucine (YNB-UL) and incubated overnight at 30 °C. The overnight cultures were then diluted to 40,000 cells/mL (OD_600_ ≈ 0.02) in a selective medium without uracil, leucine, and histidine (YNB-ULH). For the determination of constitutive activity, 50 μL of yeast cells and 150 μL of YNB-ULH medium containing different concentrations of 3-AT and 0.8 IU/mL ADA were then added to each well. To obtain concentration–growth curves, 2 μL of various concentrations of ligands, 50 μL of yeast cells, and 150 μL of YNB-ULH medium containing 7 mM 3-AT and 0.8 IU/mL ADA were then added to each well. After incubation at 30 °C for 35 h in a Genios plate reader (TECAN, Zürich, Switzerland) with shaking for 1 min at 300 rpm every 10 min, the optical density was measured at a wavelength of 595 nm, which represented the level of yeast cell growth.

### 4.6. Cell Culture, Transient Transfection, and Membrane Preparation

Chinese hamster ovary (CHO) cells were cultured at 37 °C in 5% CO_2_ in a Dulbecco’s Modified Eagle’s Medium/Ham’s F12 (1:1, DMEM/F12) containing 10% bovine calf serum, streptomycin (50 μg/mL), and penicillin (50 IU/mL). Cells were grown until 80–90% confluency and subcultured twice weekly. 

Transient transfection of CHO cells with wild type or mutated hA_1_AR plasmid constructs was performed using a polyethylenimine (PEI) method [57]. Cells were seeded in 10-cm culture dishes to achieve 50–60% confluency 24 h prior to transfection. On the day of transfection, cells were transfected with a PEI: DNA ratio of 3:1 and a plasmid DNA amount of 10 μg/dish. Moreover, 24 h post-transfection, the medium was refreshed, and 48 h after transfection, cells were collected and membranes were prepared as previously described [58]. Membranes were aliquoted in 250 or 100 μL and stored at −80 °C. Membrane protein concentrations were determined using the BCA method [59].

### 4.7. Enzyme-Linked Immunosorbent Assay

The ELISA experiments were performed with some modifications from a previously published procedure [60]. Moreover, 24 h after transfection, cells were seeded in a 96-well plate with a density of 10^6^ cells per well; 48 h post-transfection, the cells were fixed with 4% formaldehyde and blocked with 2% bovine serum albumin (BSA) (Sigma-Aldrich Chemie N.V., Zwijndrecht, The Netherlands) in Tris-buffered saline (TBS) for 1 h. Then, the cells were incubated with rabbit anti-HA tag primary antibody (1:2500) in TBST (0.05% Tween 20 in TBS) overnight at 4 °C. The cells were washed 3 times in TBST and incubated with the goat anti-rabbit IgG HRP secondary antibody (1:6000) for 1 h at RT. After removing the secondary antibody and washing the cells with TBS, 3,3′,5,5′-tetramethyl-benzidine (TMB) was added and incubated for 10 min in the dark. The reaction was stopped with 1 M H_3_PO4, and absorbance was read at 450 nm using a Wallac EnVision 2104 Multilabel reader (PerkinElmer). 

### 4.8. Radioligand Displacement Assay

The displacement assays were performed as described previously [27]. Briefly, to each well the following was added: 25 µL cell membrane suspension, 25 µL of 1.6 nM radioligand [^3^H]DPCPX, 25 µL of assay buffer (50 mM Tris-HCl, pH 7.4), and 25 µL of six increasing concentrations of DPCPX (10^−11^ to 10^−6^ M) or CPA (10^−10^ to 10^−5^ M), all dissolved in assay buffer. Note, the number of cell membranes (10–25 µg) was adjusted to obtain (approximately) a 1500 DPM assay window for each mutant. Nonspecific binding was determined in the presence of 10^−4^ M CPA and represented less than 10% of the total binding. For homologous competition assays, radioligand displacement experiments were performed in the presence of 3 different concentrations of [^3^H]DPCPX (1.6, 4.5, and 10 nM) as well as 6 increasing concentrations of DPCPX (10^−11^ to 10^−6^ M). Incubations were terminated after 1 h at 25 °C by rapid vacuum filtration through GF/B filter plates (PerkinElmer, Groningen, Netherlands) using a Perkin Elmer Filtermate-harvester. Afterward, filter plates were washed ten times with ice-cold buffer (50 mM Tris-HCl, pH 7.4) and dried at 55 °C for 30 min. After the addition of 25 µL per well of the Microscint scintillation cocktail (PerkinElmer, Groningen, The Netherlands), the filter-bound radioactivity was measured by scintillation spectrometry in a Microbeta2^®^ 2450 microplate counter (PerkinElmer).

### 4.9. [^35^S]GTPγS Binding Assay

[^35^S]GTPγS binding assays were adapted from a previously published method [27]. Membrane aliquots containing 15 µg protein were incubated with a total volume of 80 µL of assay buffer (50 mM Tris-HCl buffer, 5 mM MgCl_2_, 1 mM EDTA, 100 mM NaCl, 0.05% BSA, and 1 mM DTT pH 7.4 supplemented with 10 μM GDP and 10 µg saponin) and 9 increasing concentrations of CPA (10^−11^ to 10^−6^ M) or 9 increasing concentrations of DPCPX (10^−11^ to 10^−6^ M) in the presence of a fixed concentration (EC_80_ for wild type or mutant hA_1_ARs) of CPA for 30 min at 4 °C. Then, 20 µL of [^35^S]GTPγS (final concentration of 0.3 nM) was added to each well, followed by 90 min of incubation at 25 °C. Incubations were terminated and filter-bound radioactivity was measured as described above.

### 4.10. Modelling

Structures of the A_1_AR in the inactive (PDB: 5UEN) [24] and active (PDB: 6D9H) states [23] and the inactive state of the A_2A_AR (PDB: 4EIY) [38] were retrieved from the PDB. Missing side chains and loop regions were added using the GPCR-ModSim web server [61]. All structures were aligned to the inactive A_1_AR, and figures were generated using the PyMOL Molecular Graphics System version 2.0 (Schrödinger, LLC., New York, NY, USA).

### 4.11. Data Analysis

All experimental data were analyzed by GraphPad Prism 7.0 or 8.0 (GraphPad Software Inc., San Diego, CA, USA). Data from yeast liquid growth and [^35^S]GTPγS binding assays were analyzed by non-linear regression using “log (agonist) vs. response (three parameters)” or “log (inhibitor) vs. response (three parameters)” to obtain potency (EC_50_), inhibitory potency (IC_50_), and efficacy (E_max_ or I_max_) values. The radioligand displacement curves were obtained from a statistically preferred one-site or two-site binding model. pK_i_ values were calculated from pIC_50_ values using the Cheng–Prusoff equation, where K_D_ values were obtained from the homologous competition assays from this study and calculated by non-linear regression using “one site–homologous” [62].

## 5. Conclusions

In conclusion, 13 cancer-induced somatic mutations located at the 7-transmembrane domain of the adenosine A_1_ receptor were retrieved from TCGA and characterized in a robust yeast system. Moreover, two CAMs (H78L^3.23^ and S246T^6.47^), one LFM (L134F^4.52^), and two CIMs (W188C^5.46^ and G279S^7.44^) were also investigated in mammalian cells. The yeast system is a simplified, suitable, rapid, and accurate method for initial mutation screening that enables us to identify mutations with a dramatic effect on receptor activation. However, the current study shows that this system is best used for receptor mutations on the extracellular side, ligand-binding pocket, or pointing inwards from the membrane. Based on the results of this study, follow-up studies focusing on the effects of these mutations on other G protein coupling pathways, as well as in a disease-relevant system, are warranted to further investigate the effect of these hA_1_AR mutations in cell proliferation and migration, and eventually in cancer progression. Taken together, this study will enrich our understanding of the largely undefined role of hA_1_AR in cancer progression, which may eventually improve cancer therapies. 

## Figures and Tables

**Table 1:** List of cancer-related somatic mutations identified from different cancer types.

Mutations	Cancer Types	Occurrence
A20T^1.43^	Colon adenocarcinoma	1
A52V^2.47^	Breast invasive carcinoma	1
D55V^2.50^	Breast invasive carcinoma	1
D55G^2.50^	Colon adenocarcinoma	1
H78L^3.23^	Lung adenocarcinoma	1
P86L^3.31^	Rectum adenocarcinoma	1
R122Q^4.40^	Colon adenocarcinoma	1
L134F^4.52^	Lung squamous cell carcinoma	1
W188C^5.46^	Colon adenocarcinoma	1
S246T^6.47^	Breast invasive carcinoma	1
T257P^6.58^	Lung adenocarcinoma	1
S267I^7.32^	Colon adenocarcinoma	1
G279S^7.44^	Colon adenocarcinoma	1

**Table 2:** Agonist (CPA)-induced receptor activation of wild type and mutant hA_1_ARs in yeast liquid growth assays.

Mutation	Basal ^a^	pEC_50_ (−log M)	E_max_^a^	Type ^b^
Wild type	1.00 ± 0.08	9.30 ± 0.08	4.83 ± 0.30	-
A20T^1.43^	0.68 ± 0.14	9.24 ± 0.08	4.23 ± 0.60	CIM
A52V^2.47^	0.24 ± 0.02 ***	6.68 ± 0.09 ****	1.86 ± 0.14 **	CIM
D55V^2.50^	0.24 ± 0.04 ***	ND	ND	LFM
D55G^2.50^	0.50 ± 0.06 **	ND	ND	LFM
H78L^3.23^	4.48 ± 0.12 ****	ND	4.15 ± 0.17	CAM
P86L^3.31^	0.28 ± 0.03 **	ND	ND	LFM
R122Q^4.40^	0.57 ± 0.22	9.04 ± 0.14	4.67 ± 0.22	CIM
L134F^4.52^	0.29 ± 0.04 **	ND	ND	LFM
W188C^5.46^	0.32 ± 0.02 **	8.21 ± 0.10 **	4.35 ± 0.10	CIM
S246T^6.47^	1.95 ± 0.27 *	9.42 ± 0.33	4.81 ± 0.26	CAM
T257P^6.58^	0.24 ± 0.01 *	ND	ND	LFM
S267I^7.32^	0.28 ± 0.01 *	ND	ND	LFM
G279S^7.44^	0.33 ± 0.12 *	9.27 ± 0.09	4.96 ± 0.38	CIM

Mutations are indicated using the numbering of the hA1AR amino acid sequence as well according to the Ballesteros and Weinstein GPCR numbering system [22]. All values are shown as mean ± SEM obtained from at least three individual experiments performed in duplicate. ^a^ Values were calculated as ratio over basal activity of wild type hA1AR. ^b^ Typing of the mutants was done according to their constitutive (in)activity and agonist-induced receptor activation. * *p* < 0.05; ** *p* < 0.01; *** *p* < 0.001; **** *p* < 0.0001 compared to wild type hA1AR, determined by a two-tailed unpaired Student’s t-test. ND: not detectable, CAM: constitutively active mutant, CIM: constitutively inactive mutant, LFM: loss of function mutant.

**Table 3:** Affinity and B_max_ values of [^3^H]DPCPX and binding affinity of CPA on wild type and mutant hA_1_ARs.

	[^3^H]DPCPX ^a^		CPA		
Mutation	pK_D_	B_max_ (pmol/mg)	pK_i_ (High)	pK_i_ (Low)	Fraction (High)
Wild type	8.36 ± 0.03	1.81 ± 0.14	9.24 ± 0.26	6.76 ± 0.05	0.15 ± 0.03
H78L3.23	8.46 ± 0.03	0.17 ± 0.01 **	8.97 ± 0.35	6.83 ± 0.09	0.33 ± 0.04
L134F4.52	8.06 ± 0.08 **	3.74 ± 0.65 **	8.38 ± 0.29	6.26 ± 0.11 **	0.34 ± 0.03
W188C5.46	8.42 ± 0.03	1.87 ± 0.12	8.02 ± 0.16 *	6.15 ± 0.01 ***	0.29 ± 0.01
S246T6.47	8.44 ± 0.05	0.11 ± 0.01 **	8.98 ± 0.16	7.19 ± 0.08 **	0.26 ± 0.03
G279S7.44	8.62 ± 0.06 *	2.11 ± 0.07	8.74 ± 0.48	6.78 ± 0.06	0.17 ± 0.04

All values are shown as mean ± SEM obtained from at least three individual experiments performed in duplicate. ^a^ Values obtained from homologous displacement of ~1.6, 4.5, and 10 nM [^3^H]DPCPX from transiently transfected wild type and mutant CHO-hA_1_AR membranes at 25 °C. * *p* < 0.05; ** *p* < 0.01; *** *p* < 0.001 compared to wild type hA_1_AR, determined by one-way ANOVA with Dunnett’s post-test.

**Table 4:** Potency and efficacy values of CPA and DPCPX in [^35^S]GTPγS binding assays on wild type and mutant hA_1_ARs.

		CPA		DPCPX	
Mutation	Basal ^a^	pEC_50_ (−log M)	E_max_^a^	pIC50 (−log M)	I_max_^b^
Wild type	1.00 ± 0.09	8.98 ± 0.08	1.48 ± 0.13	8.09 ± 0.16	0.67 ± 0.05
H78L3.23	1.24 ± 0.10	9.09 ± 0.12	1.40 ± 0.10	8.19 ± 0.25	0.83 ± 0.03
L134F4.52	1.12 ± 0.17	9.08 ± 0.16	1.48 ± 0.24	8.14 ± 0.23	0.68 ± 0.01
W188C5.46	1.21 ± 0.06	8.28 ± 0.10 *	1.94 ± 0.02	7.87± 0.25	0.74 ± 0.03
S246T6.47	1.08 ± 0.10	9.44 ± 0.22	1.21 ± 0.10	8.44 ± 0.10	0.70 ± 0.05
G279S7.44	1.17 ± 0.13	8.69 ± 0.10	1.57 ± 0.20	8.23 ± 0.06	0.65 ± 0.08

All values are shown as mean ± SEM obtained from at least three individual experiments performed in duplicate. ^a^ Values were calculated as ratio over basal activity of wild type hA_1_AR. ^b^ Values were calculated as ratio over basal activity of wild type or mutant hA_1_AR. * *p* < 0.05 compared to wild type hA_1_AR, determined by one-way ANOVA with Dunnett’s post-test.

## Data Availability

The data that support the findings of this study are available upon reasonable request from the corresponding author.

## Sample Availability

Samples of the compounds were bought from official suppliers as described.
